# Supplementary material for: Synergistic effects of selenium nanoparticles and LED light on enhancement of secondary metabolites in sandalwood (Santalum album) plants through in-vitro callus culturing technique
Source: PeerJ. 2024 Sep 26;12:e18106. doi: 10.7717/peerj.18106 (PMC11439390; doi:10.7717/peerj.18106)
Supplement: Supplemental Information 1 [file peerj-12-18106-s001.pdf]

XLSTAT 2014.5.03 - Principal Component Analysis (PCA) - on 31/05/2023 at 07:36:56
Observations/variables table: Workbook = sandalwood.xlsx / Sheet = Sheet2 / Range = Sheet2!D2:$
PCA type: Spearman
Type of biplot: Distance biplot / Coefficient = Automatic

Summary statistics:

Variable	)bservation	with missin	thout missi	Minimum	Maximum	Mean	td. deviation
CFW	48	0	48	101.0000	150.0000	121.3298	15.7164
CDW	48	0	48	21.0000	49.0000	31.3792	7.5762
CMC	48	0	48	47.5000	85.0000	63.2292	11.7063
nosb/c	48	0	48	14.3000	24.8700	19.6038	2.6946
TFC	48	0	48	0.0230	0.0980	0.0563	0.0226
TPC	48	0	48	10.3400	19.2200	14.0711	2.3218
TSC	48	0	48	3.7800	9.2300	6.1992	1.7574
TTTC	48	0	48	6.1100	14.9900	9.8411	2.3218
TiAC	48	0	48	0.1230	0.6040	0.3274	0.1690
t.flavanols	48	0	48	2.1290	7.5790	4.5482	1.7574
Tctc	48	0	48	0.5700	3.4900	1.6394	0.9368
PAL	48	0	48	2.7340	6.4730	4.0759	1.2951
SOD	48	0	48	1.2040	4.9620	2.4788	1.4276
POD	48	0	48	1.5310	4.5740	2.5315	1.1046
CAT	48	0	48	0.5010	3.1630	1.4135	1.0146
dpph	48	0	48	23.0000	87.0000	53.1667	18.3156
toco ug/g F	48	0	48	3.3200	4.9700	4.0393	0.4323
antho mg/	48	0	48	3.0800	6.0000	4.1494	0.9368

Correlation matrix (Spearman):

Variables	CFW	CDW	CMC	nosb/c	TFC	TPC	TSC	TTTC
CFW	**1**	0.9815	0.9833	0.9822	0.9954	0.9900	0.9821	0.9900
CDW	0.9815	**1**	0.9944	0.9439	0.9829	0.9947	0.9450	0.9947
CMC	0.9833	0.9944	**1**	0.9468	0.9832	0.9971	0.9476	0.9971
nosb/c	0.9822	0.9439	0.9468	**1**	0.9820	0.9586	0.9928	0.9586
TFC	0.9954	0.9829	0.9832	0.9820	**1**	0.9900	0.9803	0.9900
TPC	0.9900	0.9947	0.9971	0.9586	0.9900	**1**	0.9576	1.0000
TSC	0.9821	0.9450	0.9476	0.9928	0.9803	0.9576	**1**	0.9576
TTTC	0.9900	0.9947	0.9971	0.9586	0.9900	1.0000	0.9576	**1**
TiAC	0.9936	0.9879	0.9901	0.9724	0.9948	0.9950	0.9714	0.9950
t.flavanols	0.9821	0.9450	0.9476	0.9928	0.9803	0.9576	1.0000	0.9576
Tctc	0.9873	0.9662	0.9659	0.9851	0.9911	0.9746	0.9866	0.9746
PAL	0.9673	0.9699	0.9716	0.9480	0.9722	0.9771	0.9372	0.9771
SOD	0.9230	0.9038	0.9001	0.9413	0.9300	0.9117	0.9273	0.9117
POD	0.9482	0.9207	0.9184	0.9637	0.9513	0.9306	0.9537	0.9306

CAT	0.9322	0.9179	0.9154	0.9453	0.9415	0.9274	0.9324	0.9274
dpph	0.9830	0.9578	0.9580	0.9878	0.9867	0.9675	0.9901	0.9675
toco ug/g F	0.9507	0.9106	0.9159	0.9787	0.9528	0.9265	0.9710	0.9265
antho mg/	0.9873	0.9662	0.9659	0.9851	0.9911	0.9746	0.9866	0.9746

Principal Component Analysis:

Eigenvalues:

	F1	F2	F3	F4	F5	F6	F7	F8
Eigenvalue	17.3740	0.3229	0.1787	0.0313	0.0231	0.0155	0.0127	0.0100
Variability	96.5224	1.7940	0.9928	0.1740	0.1284	0.0862	0.0707	0.0555
Cumulative	96.5224	98.3165	99.3093	99.4832	99.6117	99.6979	99.7686	99.8242

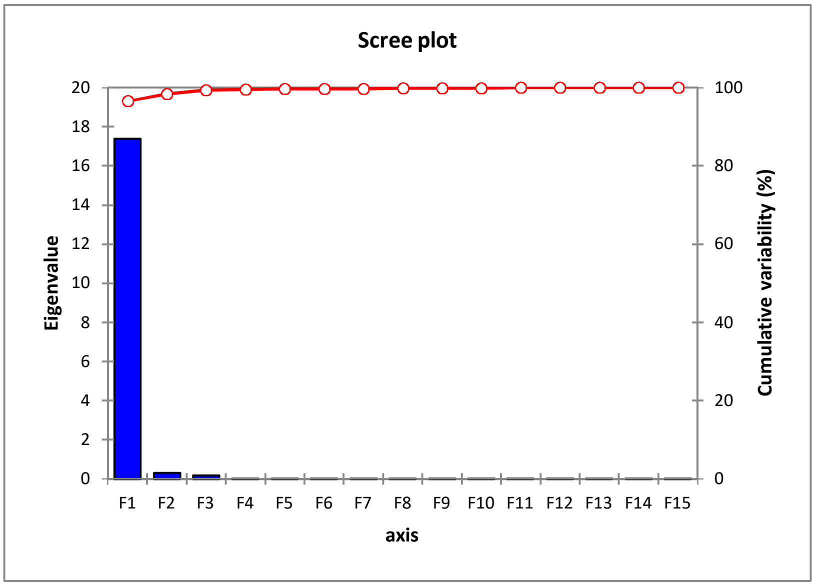

Eigenvectors:

	F1	F2	F3	F4	F5	F6	F7	F8
CFW	0.2383	-0.1485	-0.0895	0.1327	-0.0882	-0.3114	0.1209	-0.2694
CDW	0.2345	-0.3081	0.1989	-0.1366	-0.2871	0.0911	0.2119	0.1311
CMC	0.2347	-0.3180	0.1730	0.1089	-0.2153	0.2840	0.0837	0.0169
nosb/c	0.2370	0.1000	-0.2762	0.2745	0.0144	-0.2353	-0.2476	-0.1450
TFC	0.2387	-0.1261	-0.0488	-0.0549	0.0833	-0.1233	-0.1261	-0.1985
TPC	0.2365	-0.2686	0.1401	0.0670	-0.0898	0.0634	0.0168	0.0072
TSC	0.2363	0.0503	-0.3764	0.1223	-0.0580	-0.1286	0.1115	0.4547
TTTC	0.2365	-0.2686	0.1401	0.0670	-0.0898	0.0634	0.0168	0.0072
TiAC	0.2379	-0.1972	0.0245	-0.0282	0.0442	-0.1001	-0.1142	-0.0896
t.flavanols	0.2363	0.0503	-0.3764	0.1223	-0.0580	-0.1286	0.1115	0.4547
Tctc	0.2385	0.0012	-0.1442	-0.4032	0.2749	0.0458	-0.0369	-0.1674

PAL	0.2346	-0.0489	0.4011	0.3459	0.5534	-0.1958	-0.2748	0.1374
SOD	0.2290	0.4305	0.3037	-0.2052	-0.5403	-0.2045	-0.5012	0.0545
POD	0.2332	0.3620	0.1494	-0.0187	-0.0533	-0.3033	0.6478	-0.3140
CAT	0.2312	0.3615	0.3433	-0.1388	0.2773	0.2294	0.2068	0.3895
dpph	0.2373	0.0125	-0.2647	-0.3197	-0.0116	0.3078	-0.1577	0.0248
toco ug/g F	0.2332	0.3438	-0.1256	0.4745	-0.0480	0.6072	-0.0319	-0.3146
antho mg/:	0.2385	0.0012	-0.1442	-0.4032	0.2749	0.0458	-0.0369	-0.1674

Factor loadings:

	F1	F2	F3	F4	F5	F6	F7	F8
CFW	0.9931	-0.0844	-0.0378	0.0235	-0.0134	-0.0388	0.0136	-0.0269
CDW	0.9775	-0.1751	0.0841	-0.0242	-0.0437	0.0113	0.0239	0.0131
CMC	0.9784	-0.1807	0.0731	0.0193	-0.0327	0.0354	0.0094	0.0017
nosb/c	0.9877	0.0568	-0.1167	0.0486	0.0022	-0.0293	-0.0279	-0.0145
TFC	0.9951	-0.0716	-0.0206	-0.0097	0.0127	-0.0154	-0.0142	-0.0198
TPC	0.9858	-0.1527	0.0592	0.0118	-0.0137	0.0079	0.0019	0.0007
TSC	0.9851	0.0286	-0.1591	0.0216	-0.0088	-0.0160	0.0126	0.0455
TTTC	0.9858	-0.1527	0.0592	0.0118	-0.0137	0.0079	0.0019	0.0007
TiAC	0.9915	-0.1121	0.0104	-0.0050	0.0067	-0.0125	-0.0129	-0.0090
t.flavanols	0.9851	0.0286	-0.1591	0.0216	-0.0088	-0.0160	0.0126	0.0455
Tctc	0.9940	0.0007	-0.0610	-0.0713	0.0418	0.0057	-0.0042	-0.0167
PAL	0.9779	-0.0278	0.1696	0.0612	0.0841	-0.0244	-0.0310	0.0137
SOD	0.9547	0.2446	0.1284	-0.0363	-0.0822	-0.0255	-0.0565	0.0054
POD	0.9721	0.2057	0.0632	-0.0033	-0.0081	-0.0378	0.0731	-0.0314
CAT	0.9639	0.2054	0.1451	-0.0246	0.0422	0.0286	0.0233	0.0389
dpph	0.9893	0.0071	-0.1119	-0.0566	-0.0018	0.0383	-0.0178	0.0025
toco ug/g F	0.9719	0.1954	-0.0531	0.0840	-0.0073	0.0756	-0.0036	-0.0315
antho mg/:	0.9940	0.0007	-0.0610	-0.0713	0.0418	0.0057	-0.0042	-0.0167

Correlations between variables and factors:

	F1	F2	F3	F4	F5	F6	F7	F8
CFW	0.9931	-0.0844	-0.0378	0.0235	-0.0134	-0.0388	0.0136	-0.0269
CDW	0.9775	-0.1751	0.0841	-0.0242	-0.0437	0.0113	0.0239	0.0131
CMC	0.9784	-0.1807	0.0731	0.0193	-0.0327	0.0354	0.0094	0.0017
nosb/c	0.9877	0.0568	-0.1167	0.0486	0.0022	-0.0293	-0.0279	-0.0145
TFC	0.9951	-0.0716	-0.0206	-0.0097	0.0127	-0.0154	-0.0142	-0.0198
TPC	0.9858	-0.1527	0.0592	0.0118	-0.0137	0.0079	0.0019	0.0007
TSC	0.9851	0.0286	-0.1591	0.0216	-0.0088	-0.0160	0.0126	0.0455
TTTC	0.9858	-0.1527	0.0592	0.0118	-0.0137	0.0079	0.0019	0.0007
TiAC	0.9915	-0.1121	0.0104	-0.0050	0.0067	-0.0125	-0.0129	-0.0090
t.flavanols	0.9851	0.0286	-0.1591	0.0216	-0.0088	-0.0160	0.0126	0.0455
Tctc	0.9940	0.0007	-0.0610	-0.0713	0.0418	0.0057	-0.0042	-0.0167
PAL	0.9779	-0.0278	0.1696	0.0612	0.0841	-0.0244	-0.0310	0.0137

SOD	0.9547	0.2446	0.1284	-0.0363	-0.0822	-0.0255	-0.0565	0.0054
POD	0.9721	0.2057	0.0632	-0.0033	-0.0081	-0.0378	0.0731	-0.0314
CAT	0.9639	0.2054	0.1451	-0.0246	0.0422	0.0286	0.0233	0.0389
dpph	0.9893	0.0071	-0.1119	-0.0566	-0.0018	0.0383	-0.0178	0.0025
toco ug/g F	0.9719	0.1954	-0.0531	0.0840	-0.0073	0.0756	-0.0036	-0.0315
antho mg/	0.9940	0.0007	-0.0610	-0.0713	0.0418	0.0057	-0.0042	-0.0167

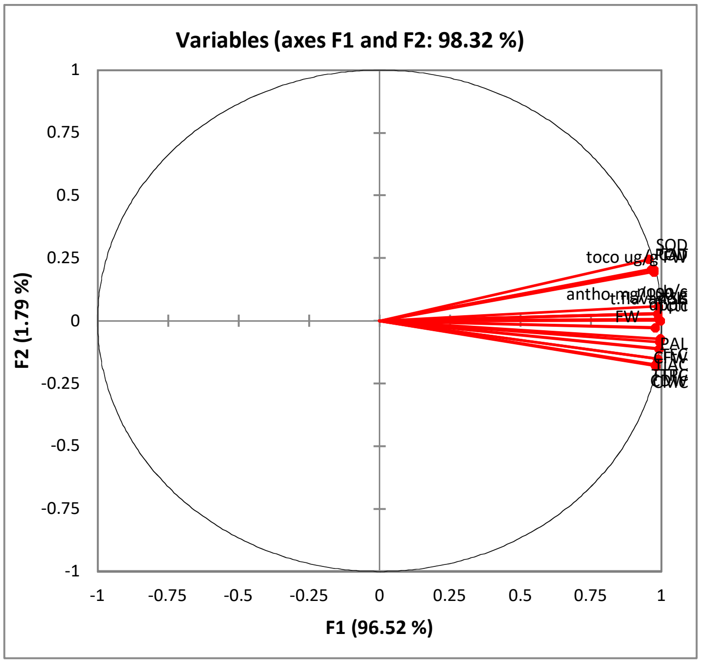

Contribution of the variables (%):

	F1	F2	F3	F4	F5	F6	F7	F8
CFW	5.6765	2.2057	0.8004	1.7601	0.7782	9.6990	1.4625	7.2587
CDW	5.4994	9.4924	3.9551	1.8651	8.2419	0.8296	4.4885	1.7196
CMC	5.5093	10.1099	2.9932	1.1858	4.6344	8.0633	0.6999	0.0285
nosb/c	5.6151	1.0006	7.6267	7.5341	0.0207	5.5358	6.1311	2.1023
TFC	5.6999	1.5897	0.2377	0.3013	0.6933	1.5204	1.5906	3.9414
TPC	5.5936	7.2160	1.9618	0.4483	0.8070	0.4016	0.0281	0.0051
TSC	5.5860	0.2535	14.1673	1.4961	0.3361	1.6533	1.2440	20.6772
TTTC	5.5936	7.2160	1.9618	0.4483	0.8070	0.4016	0.0281	0.0051
TiAC	5.6587	3.8891	0.0600	0.0793	0.1953	1.0022	1.3032	0.8034
t.flavanols	5.5860	0.2535	14.1673	1.4961	0.3361	1.6533	1.2440	20.6772
Tctc	5.6874	0.0001	2.0793	16.2575	7.5581	0.2100	0.1364	2.8035
PAL	5.5044	0.2388	16.0889	11.9631	30.6229	3.8354	7.5542	1.8877
SOD	5.2459	18.5291	9.2217	4.2122	29.1922	4.1811	25.1172	0.2969
POD	5.4396	13.1051	2.2322	0.0350	0.2840	9.2017	41.9697	9.8596
CAT	5.3472	13.0661	11.7822	1.9255	7.6907	5.2610	4.2763	15.1720
dpph	5.6333	0.0156	7.0086	10.2209	0.0134	9.4761	2.4883	0.0614
toco ug/g F	5.4367	11.8186	1.5766	22.5139	0.2306	36.8646	0.1015	9.8968

antho mg/	5.6874	0.0001	2.0793	16.2575	7.5581	0.2100	0.1364	2.8035

Squared cosines of the variables:

	F1	F2	F3	F4	F5	F6	F7	F8
CFW	**0.9862**	0.0071	0.0014	0.0006	0.0002	0.0015	0.0002	0.0007
CDW	**0.9555**	0.0307	0.0071	0.0006	0.0019	0.0001	0.0006	0.0002
CMC	**0.9572**	0.0326	0.0053	0.0004	0.0011	0.0013	0.0001	0.0000
nosb/c	**0.9756**	0.0032	0.0136	0.0024	0.0000	0.0009	0.0008	0.0002
TFC	**0.9903**	0.0051	0.0004	0.0001	0.0002	0.0002	0.0002	0.0004
TPC	**0.9718**	0.0233	0.0035	0.0001	0.0002	0.0001	0.0000	0.0000
TSC	**0.9705**	0.0008	0.0253	0.0005	0.0001	0.0003	0.0002	0.0021
TTTC	**0.9718**	0.0233	0.0035	0.0001	0.0002	0.0001	0.0000	0.0000
TiAC	**0.9831**	0.0126	0.0001	0.0000	0.0000	0.0002	0.0002	0.0001
t.flavanols	**0.9705**	0.0008	0.0253	0.0005	0.0001	0.0003	0.0002	0.0021
Tctc	**0.9881**	0.0000	0.0037	0.0051	0.0017	0.0000	0.0000	0.0003
PAL	**0.9563**	0.0008	0.0288	0.0037	0.0071	0.0006	0.0010	0.0002
SOD	**0.9114**	0.0598	0.0165	0.0013	0.0067	0.0006	0.0032	0.0000
POD	**0.9451**	0.0423	0.0040	0.0000	0.0001	0.0014	0.0053	0.0010
CAT	**0.9290**	0.0422	0.0211	0.0006	0.0018	0.0008	0.0005	0.0015
dpph	**0.9787**	0.0001	0.0125	0.0032	0.0000	0.0015	0.0003	0.0000
toco ug/g F	**0.9446**	0.0382	0.0028	0.0070	0.0001	0.0057	0.0000	0.0010
antho mg/	**0.9881**	0.0000	0.0037	0.0051	0.0017	0.0000	0.0000	0.0003

Values in bold correspond for each variable to the factor for which the squared cosine is the largest

Factor scores:

Observatior	F1	F2	F3	F4	F5	F6	F7	F8
Obs1	-5.6344	0.4236	0.6269	-0.4583	0.1764	-0.2447	0.2656	0.1420
Obs2	-5.4445	0.2567	0.6406	-0.4727	0.1888	-0.4297	0.2415	0.1074
Obs3	-5.1849	0.2373	0.5626	-0.4576	0.1207	-0.1784	0.2422	0.0043
Obs4	-4.2765	-0.2364	0.5629	-0.1448	-0.0768	-0.0763	0.1621	0.0064
Obs5	-4.1663	-0.1114	0.4382	-0.0395	-0.0118	0.0342	0.0651	-0.1251
Obs6	-3.9851	-0.2094	0.3687	-0.0840	-0.0301	-0.0089	0.0183	-0.1259
Obs7	-3.5513	-0.4339	0.3708	-0.1566	-0.1043	0.1695	0.0570	-0.1944
Obs8	-3.3033	-0.4587	0.2796	-0.0571	-0.1107	0.1850	0.0173	-0.2101
Obs9	-3.2172	-0.5224	0.2663	-0.1246	-0.0866	0.1593	-0.0011	-0.1370
Obs10	-2.8944	-0.5278	0.0900	-0.0457	-0.1318	0.2250	-0.0028	-0.0554
Obs11	-2.8370	-0.5917	0.0820	-0.0406	-0.1197	0.1164	-0.0129	-0.0285
Obs12	-2.6571	-0.6204	-0.0178	-0.0570	-0.1326	0.1447	-0.0413	-0.0536
Obs13	-4.5425	0.6824	0.2262	0.1610	0.2661	0.1115	-0.1340	-0.2687
Obs14	-4.4207	0.4926	0.3489	0.0437	0.1887	0.0848	-0.0072	-0.2414
Obs15	-4.2240	0.5002	0.2312	0.1615	0.1964	0.0732	-0.0728	-0.3068
Obs16	-2.0931	-0.5488	-0.2221	0.0941	-0.1816	0.2388	-0.0798	-0.0124
Obs17	-1.9134	-0.5479	-0.3012	0.0625	-0.2962	0.2240	-0.0613	-0.0817

Obs18	-1.6825	-0.5763	-0.3777	0.1482	-0.3599	0.2884	-0.1150	0.0369
Obs19	-0.7421	-0.1410	-0.0586	0.2806	-0.1853	-0.1353	0.1991	0.0288
Obs20	-0.4854	-0.2161	-0.0930	0.2845	-0.1375	-0.1133	0.2108	-0.0411
Obs21	-0.3548	-0.2778	-0.1391	0.4285	-0.0850	-0.1920	0.2543	-0.0705
Obs22	0.8168	0.8292	0.4331	0.0604	-0.2798	-0.2518	-0.0314	0.6946
Obs23	0.8104	0.7625	0.4198	0.1105	-0.4568	-0.1107	-0.1877	0.5903
Obs24	1.0332	0.6970	0.3220	0.0373	-0.3792	-0.0829	-0.1810	0.3488
Obs25	-3.6285	0.3600	-0.4963	0.0990	0.0755	0.1107	-0.0988	0.0078
Obs26	-3.5637	0.2529	-0.4768	-0.2288	0.1481	0.0689	-0.0592	0.1571
Obs27	-3.3602	0.2713	-0.6249	-0.0426	0.0882	-0.0344	-0.1743	0.0450
Obs28	0.9048	-0.5680	-0.7258	0.3724	0.1435	0.0616	-0.3938	0.1351
Obs29	1.0316	-0.7477	-0.6539	0.2199	0.0471	-0.1366	-0.3165	0.1394
Obs30	1.2709	-0.5808	-0.8282	0.1901	0.0268	0.1340	-0.3591	-0.0130
Obs31	2.8196	0.9638	0.3868	-0.1345	-0.2689	-0.4210	-0.0378	0.1992
Obs32	3.0906	0.8694	0.2470	-0.2324	-0.2806	-0.5153	-0.0675	0.0764
Obs33	3.2322	0.7855	0.1449	-0.2827	-0.3407	-0.4150	-0.1454	-0.0451
Obs34	3.2076	-0.1148	-0.5624	0.2057	0.4549	-0.0232	0.2919	0.0923
Obs35	3.4602	-0.1588	-0.4142	0.1284	0.4286	0.0402	0.3441	0.0695
Obs36	3.5142	-0.1502	-0.5533	-0.0031	0.3904	0.1435	0.2708	-0.0661
Obs37	-2.9084	0.2281	-0.8221	0.0794	0.1007	-0.0249	-0.1438	0.1197
Obs38	-2.8230	0.1571	-0.7480	0.2385	0.0503	-0.0280	-0.1129	0.1220
Obs39	-2.7039	0.1394	-0.8145	0.3717	0.0315	-0.1115	-0.1466	0.0678
Obs40	5.3547	0.2026	0.3389	-0.3495	0.3235	0.0472	-0.0773	-0.1990
Obs41	5.6071	0.2276	0.3231	-0.1849	0.2520	0.0998	-0.0448	-0.2913
Obs42	5.7883	0.0106	0.3548	-0.1752	0.1110	0.0429	0.0536	-0.2459
Obs43	6.7092	-0.0271	0.1343	-0.0471	0.2130	0.0259	0.0014	0.0213
Obs44	6.8933	-0.1168	0.1195	-0.0722	0.1282	0.0089	0.0286	0.0712
Obs45	7.1570	-0.1414	0.0821	-0.1057	0.1138	0.0777	0.0271	-0.0084
Obs46	7.7600	-0.2107	0.1126	0.0371	0.0133	0.0809	0.0834	-0.1086
Obs47	7.9809	-0.2658	0.1966	0.1312	-0.0711	0.1656	0.1575	-0.1380
Obs48	8.1558	-0.2478	0.2196	0.0514	-0.1506	0.3711	0.1143	-0.2158

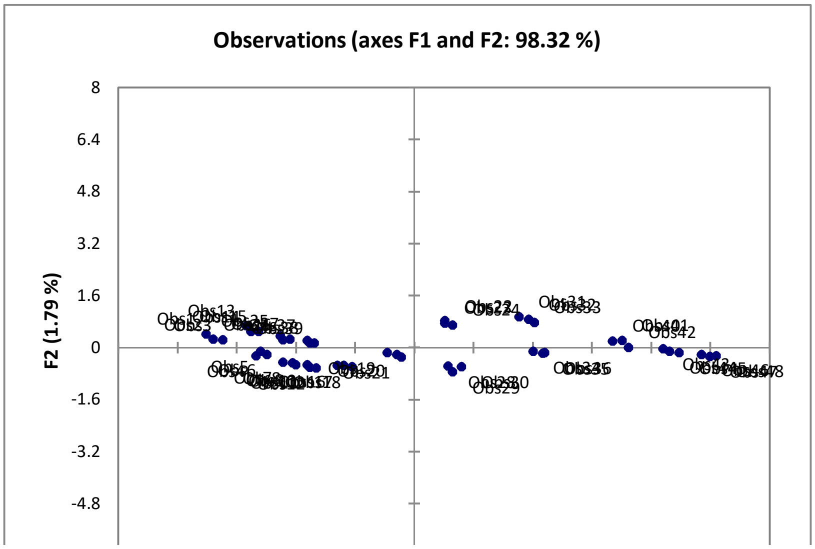

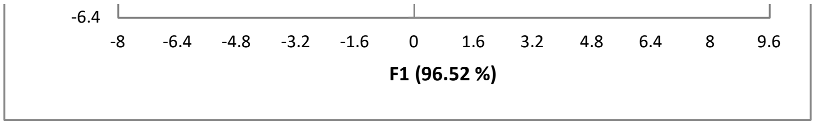

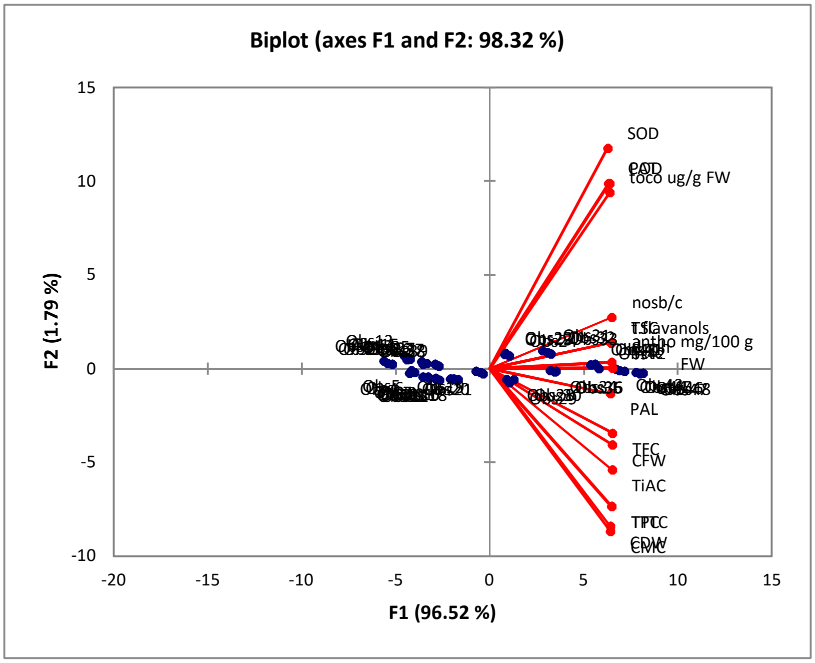

Contribution of the observations (%):

	F1	F2	F3	F4	F5	F6	F7	F8
Obs1	3.8068	1.1575	4.5821	13.9767	2.8047	8.0418	11.5439	4.2030
Obs2	3.5544	0.4250	4.7842	14.8652	3.2108	24.7946	9.5460	2.4045
Obs3	3.2236	0.3633	3.6896	13.9335	1.3135	4.2741	9.5994	0.0039
Obs4	2.1930	0.3605	3.6942	1.3945	0.5316	0.7820	4.2974	0.0086
Obs5	2.0814	0.0800	2.2382	0.1040	0.0126	0.1574	0.6933	3.2599
Obs6	1.9043	0.2829	1.5848	0.4695	0.0816	0.0106	0.0549	3.3023
Obs7	1.5123	1.2148	1.6025	1.6326	0.9807	3.8591	0.5323	7.8756
Obs8	1.3084	1.3575	0.9112	0.2167	1.1038	4.5959	0.0492	9.2052
Obs9	1.2411	1.7605	0.8267	1.0334	0.6756	3.4061	0.0002	3.9151
Obs10	1.0046	1.7970	0.0943	0.1389	1.5651	6.7980	0.0013	0.6399
Obs11	0.9651	2.2588	0.0785	0.1099	1.2917	1.8197	0.0272	0.1688
Obs12	0.8466	2.4834	0.0037	0.2162	1.5854	2.8113	0.2795	0.5985
Obs13	2.4743	3.0046	0.5966	1.7249	6.3820	1.6700	2.9366	15.0456
Obs14	2.3434	1.5653	1.4194	0.1268	3.2096	0.9662	0.0084	12.1427
Obs15	2.1395	1.6145	0.6232	1.7361	3.4750	0.7194	0.8671	19.6169
Obs16	0.5254	1.9432	0.5752	0.5886	2.9728	7.6570	1.0408	0.0322
Obs17	0.4390	1.9368	1.0577	0.2597	7.9059	6.7366	0.6157	1.3920
Obs18	0.3395	2.1427	1.6631	1.4604	11.6712	11.1684	2.1633	0.2834
Obs19	0.0660	0.1283	0.0400	5.2377	3.0951	2.4564	6.4833	0.1732
Obs20	0.0283	0.3012	0.1009	5.3840	1.7030	1.7225	7.2742	0.3518

Obs21	0.0151	0.4978	0.2255	12.2136	0.6509	4.9493	10.5801	1.0355
Obs22	0.0800	4.4360	2.1865	0.2426	7.0556	8.5090	0.1614	100.5653
Obs23	0.0788	3.7512	2.0542	0.8122	18.8013	1.6464	5.7631	72.6414
Obs24	0.1280	3.1342	1.2083	0.0926	12.9556	0.9233	5.3588	25.3591
Obs25	1.5787	0.8360	2.8715	0.6526	0.5131	1.6446	1.5980	0.0128
Obs26	1.5228	0.4126	2.6499	3.4833	1.9763	0.6381	0.5729	5.1460
Obs27	1.3539	0.4748	4.5521	0.1207	0.7013	0.1585	4.9714	0.4228
Obs28	0.0982	2.0816	6.1414	9.2247	1.8547	0.5103	25.3732	3.8048
Obs29	0.1276	3.6063	4.9850	3.2171	0.2003	2.5039	16.3881	4.0496
Obs30	0.1937	2.1761	7.9958	2.4039	0.0645	2.4090	21.0975	0.0353
Obs31	0.9533	5.9932	1.7437	1.2044	6.5176	23.7993	0.2334	8.2749
Obs32	1.1453	4.8764	0.7114	3.5935	7.0926	35.6455	0.7459	1.2155
Obs33	1.2527	3.9808	0.2446	5.3189	10.4571	23.1225	3.4600	0.4231
Obs34	1.2337	0.0850	3.6867	2.8142	18.6446	0.0722	13.9442	1.7757
Obs35	1.4357	0.1626	1.9997	1.0971	16.5556	0.2174	19.3694	1.0069
Obs36	1.4808	0.1456	3.5689	0.0006	13.7348	2.7635	12.0012	0.9111
Obs37	1.0143	0.3357	7.8784	0.4199	0.9139	0.0834	3.3849	2.9877
Obs38	0.9556	0.1591	6.5220	3.7857	0.2280	0.1052	2.0866	3.1011
Obs39	0.8767	0.1253	7.7337	9.1923	0.0894	1.6688	3.5163	0.9593
Obs40	3.4382	0.2647	1.3386	8.1252	9.4284	0.2995	0.9789	8.2513
Obs41	3.7699	0.3343	1.2169	2.2758	5.7215	1.3365	0.3288	17.6864
Obs42	4.0175	0.0007	1.4672	2.0413	1.1110	0.2474	0.4701	12.5996
Obs43	5.3976	0.0047	0.2102	0.1474	4.0884	0.0901	0.0003	0.0944
Obs44	5.6979	0.0881	0.1665	0.3470	1.4819	0.0105	0.1335	1.0557
Obs45	6.1422	0.1290	0.0785	0.7430	1.1675	0.8104	0.1206	0.0146
Obs46	7.2208	0.2863	0.1477	0.0914	0.0160	0.8786	1.1372	2.4566
Obs47	7.6376	0.4558	0.4504	1.1461	0.4550	3.6800	4.0569	3.9676
Obs48	7.9760	0.3963	0.5623	0.1755	2.0441	18.4888	2.1373	9.7089

Squared cosines of the observations:

	F1	F2	F3	F4	F5	F6	F7	F8
Obs1	**0.9582**	0.0054	0.0119	0.0063	0.0009	0.0018	0.0021	0.0006
Obs2	**0.9583**	0.0021	0.0133	0.0072	0.0012	0.0060	0.0019	0.0004
Obs3	**0.9615**	0.0020	0.0113	0.0075	0.0005	0.0011	0.0021	0.0000
Obs4	**0.9728**	0.0030	0.0169	0.0011	0.0003	0.0003	0.0014	0.0000
Obs5	**0.9799**	0.0007	0.0108	0.0001	0.0000	0.0001	0.0002	0.0009
Obs6	**0.9822**	0.0027	0.0084	0.0004	0.0001	0.0000	0.0000	0.0010
Obs7	**0.9599**	0.0143	0.0105	0.0019	0.0008	0.0022	0.0002	0.0029
Obs8	**0.9575**	0.0185	0.0069	0.0003	0.0011	0.0030	0.0000	0.0039
Obs9	**0.9508**	0.0251	0.0065	0.0014	0.0007	0.0023	0.0000	0.0017
Obs10	**0.9500**	0.0316	0.0009	0.0002	0.0020	0.0057	0.0000	0.0003
Obs11	**0.9435**	0.0410	0.0008	0.0002	0.0017	0.0016	0.0000	0.0001
Obs12	**0.9303**	0.0507	0.0000	0.0004	0.0023	0.0028	0.0002	0.0004
Obs13	**0.9650**	0.0218	0.0024	0.0012	0.0033	0.0006	0.0008	0.0034
Obs14	**0.9708**	0.0121	0.0060	0.0001	0.0018	0.0004	0.0000	0.0029

Obs15	**0.9691**	0.0136	0.0029	0.0014	0.0021	0.0003	0.0003	0.0051
Obs16	**0.8840**	0.0608	0.0100	0.0018	0.0067	0.0115	0.0013	0.0000
Obs17	**0.8425**	0.0691	0.0209	0.0009	0.0202	0.0115	0.0009	0.0015
Obs18	**0.7680**	0.0901	0.0387	0.0060	0.0351	0.0226	0.0036	0.0004
Obs19	**0.5861**	0.0212	0.0037	0.0838	0.0366	0.0195	0.0422	0.0009
Obs20	**0.3550**	0.0703	0.0130	0.1219	0.0285	0.0193	0.0670	0.0025
Obs21	0.1753	0.1075	0.0269	**0.2557**	0.0101	0.0514	0.0901	0.0069
Obs22	0.2798	**0.2884**	0.0787	0.0015	0.0328	0.0266	0.0004	0.2024
Obs23	**0.2895**	0.2564	0.0777	0.0054	0.0920	0.0054	0.0155	0.1536
Obs24	**0.4713**	0.2145	0.0458	0.0006	0.0635	0.0030	0.0145	0.0537
Obs25	**0.9656**	0.0095	0.0181	0.0007	0.0004	0.0009	0.0007	0.0000
Obs26	**0.9646**	0.0049	0.0173	0.0040	0.0017	0.0004	0.0003	0.0019
Obs27	**0.9452**	0.0062	0.0327	0.0002	0.0007	0.0001	0.0025	0.0002
Obs28	**0.3748**	0.1477	0.2412	0.0635	0.0094	0.0017	0.0710	0.0084
Obs29	**0.4455**	0.2340	0.1790	0.0202	0.0009	0.0078	0.0419	0.0081
Obs30	**0.5336**	0.1114	0.2266	0.0119	0.0002	0.0059	0.0426	0.0001
Obs31	**0.7961**	0.0930	0.0150	0.0018	0.0072	0.0178	0.0001	0.0040
Obs32	**0.8468**	0.0670	0.0054	0.0048	0.0070	0.0235	0.0004	0.0005
Obs33	**0.8703**	0.0514	0.0017	0.0067	0.0097	0.0143	0.0018	0.0002
Obs34	**0.8788**	0.0011	0.0270	0.0036	0.0177	0.0000	0.0073	0.0007
Obs35	**0.9191**	0.0019	0.0132	0.0013	0.0141	0.0001	0.0091	0.0004
Obs36	**0.9073**	0.0017	0.0225	0.0000	0.0112	0.0015	0.0054	0.0003
Obs37	**0.8984**	0.0055	0.0718	0.0007	0.0011	0.0001	0.0022	0.0015
Obs38	**0.9102**	0.0028	0.0639	0.0065	0.0003	0.0001	0.0015	0.0017
Obs39	**0.8807**	0.0023	0.0799	0.0166	0.0001	0.0015	0.0026	0.0006
Obs40	**0.9798**	0.0014	0.0039	0.0042	0.0036	0.0001	0.0002	0.0014
Obs41	**0.9820**	0.0016	0.0033	0.0011	0.0020	0.0003	0.0001	0.0027
Obs42	**0.9860**	0.0000	0.0037	0.0009	0.0004	0.0001	0.0001	0.0018
Obs43	**0.9937**	0.0000	0.0004	0.0000	0.0010	0.0000	0.0000	0.0000
Obs44	**0.9925**	0.0003	0.0003	0.0001	0.0003	0.0000	0.0000	0.0001
Obs45	**0.9931**	0.0004	0.0001	0.0002	0.0003	0.0001	0.0000	0.0000
Obs46	**0.9925**	0.0007	0.0002	0.0000	0.0000	0.0001	0.0001	0.0002
Obs47	**0.9886**	0.0011	0.0006	0.0003	0.0001	0.0004	0.0004	0.0003
Obs48	**0.9894**	0.0009	0.0007	0.0000	0.0003	0.0020	0.0002	0.0007

Values in bold correspond for each observation to the factor for which the squared cosine is the large

͵U$50 / 48 rows and 18 columns

TiAC	t.flavanols	Tctc	PAL	SOD	POD	CAT	dpph	oco ug/g FW	o mg/100 g
0.9936	0.9821	0.9873	0.9673	0.9230	0.9482	0.9322	0.9830	0.9507	0.9873
0.9879	0.9450	0.9662	0.9699	0.9038	0.9207	0.9179	0.9578	0.9106	0.9662
0.9901	0.9476	0.9659	0.9716	0.9001	0.9184	0.9154	0.9580	0.9159	0.9659
0.9724	0.9928	0.9851	0.9480	0.9413	0.9637	0.9453	0.9878	0.9787	0.9851
0.9948	0.9803	0.9911	0.9722	0.9300	0.9513	0.9415	0.9867	0.9528	0.9911
0.9950	0.9576	0.9746	0.9771	0.9117	0.9306	0.9274	0.9675	0.9265	0.9746
0.9714	1.0000	0.9866	0.9372	0.9273	0.9537	0.9324	0.9901	0.9710	0.9866
0.9950	0.9576	0.9746	0.9771	0.9117	0.9306	0.9274	0.9675	0.9265	0.9746
**1**	0.9714	0.9854	0.9741	0.9210	0.9412	0.9345	0.9782	0.9402	0.9854
0.9714	**1**	0.9866	0.9372	0.9273	0.9537	0.9324	0.9901	0.9710	0.9866
0.9854	0.9866	**1**	0.9610	0.9410	0.9624	0.9516	0.9930	0.9646	1.0000
0.9741	0.9372	0.9610	**1**	0.9422	0.9537	0.9609	0.9451	0.9387	0.9610
0.9210	0.9273	0.9410	0.9422	**1**	0.9840	0.9841	0.9335	0.9648	0.9410
0.9412	0.9537	0.9624	0.9537	0.9840	**1**	0.9868	0.9546	0.9791	0.9624

0.9345	0.9324	0.9516	0.9609	0.9841	0.9868	**1**	0.9408	0.9671	0.9516
0.9782	0.9901	0.9930	0.9451	0.9335	0.9546	0.9408	**1**	0.9661	0.9930
0.9402	0.9710	0.9646	0.9387	0.9648	0.9791	0.9671	0.9661	**1**	0.9646
0.9854	0.9866	1.0000	0.9610	0.9410	0.9624	0.9516	0.9930	0.9646	**1**

F9	F10	F11	F12	F13	F14	F15
0.0086	0.0067	0.0050	0.0036	0.0034	0.0028	0.0017
0.0479	0.0371	0.0276	0.0199	0.0187	0.0154	0.0093
99.8721	99.9092	99.9368	99.9567	99.9754	99.9907	100.0000

F9	F10	F11	F12	F13	F14	F15
0.0418	-0.0893	-0.0111	0.5531	-0.2273	-0.3860	0.4326
-0.1199	0.4607	0.5807	-0.1659	-0.0292	-0.2163	0.0030
-0.1544	-0.1982	-0.2251	-0.1753	-0.1421	0.5144	0.4882
0.4497	0.1087	0.2153	-0.4100	-0.4315	0.1718	0.0102
0.1811	0.1705	0.2441	0.4155	0.4847	0.5548	-0.0857
0.1454	-0.1124	-0.2109	0.0966	-0.1366	-0.0565	-0.4753
-0.1725	-0.0616	-0.0221	0.0362	0.0834	0.0344	-0.0490
0.1454	-0.1124	-0.2109	0.0966	-0.1366	-0.0565	-0.4753
0.1787	-0.4566	0.0644	-0.4125	0.5644	-0.3444	0.1350
-0.1725	-0.0616	-0.0221	0.0362	0.0834	0.0344	-0.0490
-0.2714	-0.1507	0.0934	-0.0377	-0.1994	0.0305	-0.0738

-0.3021	0.3057	-0.1533	-0.0561	0.0261	-0.0808	0.0669
-0.1707	-0.0741	-0.0394	0.0598	-0.0086	0.0033	-0.0137
-0.0424	0.1519	-0.2548	-0.2341	0.1279	0.0975	-0.0911
0.4627	-0.1986	0.1854	0.1790	-0.1030	0.0032	0.1556
0.2639	0.5004	-0.4924	-0.0272	0.1227	-0.1919	0.1889
-0.1950	-0.0297	0.1662	0.0802	0.1185	-0.1421	-0.0916
-0.2714	-0.1507	0.0934	-0.0377	-0.1994	0.0305	-0.0738

F9	F10	F11	F12	F13	F14	F15
0.0039	-0.0073	-0.0008	0.0331	-0.0132	-0.0203	0.0177
-0.0111	0.0377	0.0410	-0.0099	-0.0017	-0.0114	0.0001
-0.0143	-0.0162	-0.0159	-0.0105	-0.0082	0.0271	0.0199
0.0418	0.0089	0.0152	-0.0245	-0.0250	0.0090	0.0004
0.0168	0.0139	0.0172	0.0248	0.0281	0.0292	-0.0035
0.0135	-0.0092	-0.0149	0.0058	-0.0079	-0.0030	-0.0194
-0.0160	-0.0050	-0.0016	0.0022	0.0048	0.0018	-0.0020
0.0135	-0.0092	-0.0149	0.0058	-0.0079	-0.0030	-0.0194
0.0166	-0.0373	0.0045	-0.0247	0.0327	-0.0181	0.0055
-0.0160	-0.0050	-0.0016	0.0022	0.0048	0.0018	-0.0020
-0.0252	-0.0123	0.0066	-0.0023	-0.0116	0.0016	-0.0030
-0.0281	0.0250	-0.0108	-0.0034	0.0015	-0.0043	0.0027
-0.0159	-0.0061	-0.0028	0.0036	-0.0005	0.0002	-0.0006
-0.0039	0.0124	-0.0180	-0.0140	0.0074	0.0051	-0.0037
0.0430	-0.0162	0.0131	0.0107	-0.0060	0.0002	0.0064
0.0245	0.0409	-0.0347	-0.0016	0.0071	-0.0101	0.0077
-0.0181	-0.0024	0.0117	0.0048	0.0069	-0.0075	-0.0037
-0.0252	-0.0123	0.0066	-0.0023	-0.0116	0.0016	-0.0030

F9	F10	F11	F12	F13	F14	F15
0.0039	-0.0073	-0.0008	0.0331	-0.0132	-0.0203	0.0177
-0.0111	0.0377	0.0410	-0.0099	-0.0017	-0.0114	0.0001
-0.0143	-0.0162	-0.0159	-0.0105	-0.0082	0.0271	0.0199
0.0418	0.0089	0.0152	-0.0245	-0.0250	0.0090	0.0004
0.0168	0.0139	0.0172	0.0248	0.0281	0.0292	-0.0035
0.0135	-0.0092	-0.0149	0.0058	-0.0079	-0.0030	-0.0194
-0.0160	-0.0050	-0.0016	0.0022	0.0048	0.0018	-0.0020
0.0135	-0.0092	-0.0149	0.0058	-0.0079	-0.0030	-0.0194
0.0166	-0.0373	0.0045	-0.0247	0.0327	-0.0181	0.0055
-0.0160	-0.0050	-0.0016	0.0022	0.0048	0.0018	-0.0020
-0.0252	-0.0123	0.0066	-0.0023	-0.0116	0.0016	-0.0030
-0.0281	0.0250	-0.0108	-0.0034	0.0015	-0.0043	0.0027

-0.0159	-0.0061	-0.0028	0.0036	-0.0005	0.0002	-0.0006
-0.0039	0.0124	-0.0180	-0.0140	0.0074	0.0051	-0.0037
0.0430	-0.0162	0.0131	0.0107	-0.0060	0.0002	0.0064
0.0245	0.0409	-0.0347	-0.0016	0.0071	-0.0101	0.0077
-0.0181	-0.0024	0.0117	0.0048	0.0069	-0.0075	-0.0037
-0.0252	-0.0123	0.0066	-0.0023	-0.0116	0.0016	-0.0030

F9	F10	F11	F12	F13	F14	F15
0.1749	0.7973	0.0123	30.5944	5.1680	14.8965	18.7154
1.4380	21.2272	33.7247	2.7532	0.0852	4.6794	0.0009
2.3825	3.9302	5.0676	3.0736	2.0199	26.4631	23.8386
20.2215	1.1820	4.6350	16.8116	18.6225	2.9506	0.0105
3.2789	2.9067	5.9608	17.2624	23.4969	30.7851	0.7348
2.1146	1.2643	4.4499	0.9333	1.8658	0.3188	22.5918
2.9741	0.3789	0.0489	0.1310	0.6954	0.1180	0.2401
2.1146	1.2643	4.4499	0.9333	1.8658	0.3188	22.5918
3.1931	20.8475	0.4148	17.0188	31.8539	11.8592	1.8215
2.9741	0.3789	0.0489	0.1310	0.6954	0.1180	0.2401
7.3684	2.2725	0.8724	0.1420	3.9743	0.0931	0.5450
9.1252	9.3458	2.3489	0.3153	0.0683	0.6533	0.4479
2.9151	0.5490	0.1552	0.3571	0.0074	0.0011	0.0188
0.1797	2.3067	6.4934	5.4785	1.6346	0.9500	0.8302
21.4083	3.9446	3.4382	3.2056	1.0613	0.0010	2.4202
6.9654	25.0435	24.2431	0.0743	1.5057	3.6819	3.5686
3.8031	0.0884	2.7635	0.6428	1.4054	2.0189	0.8386

7.3684	2.2725	0.8724	0.1420	3.9743	0.0931	0.5450

F9	F10	F11	F12	F13	F14	F15
0.0000	0.0001	0.0000	0.0011	0.0002	0.0004	0.0003
0.0001	0.0014	0.0017	0.0001	0.0000	0.0001	0.0000
0.0002	0.0003	0.0003	0.0001	0.0001	0.0007	0.0004
0.0017	0.0001	0.0002	0.0006	0.0006	0.0001	0.0000
0.0003	0.0002	0.0003	0.0006	0.0008	0.0009	0.0000
0.0002	0.0001	0.0002	0.0000	0.0001	0.0000	0.0004
0.0003	0.0000	0.0000	0.0000	0.0000	0.0000	0.0000
0.0002	0.0001	0.0002	0.0000	0.0001	0.0000	0.0004
0.0003	0.0014	0.0000	0.0006	0.0011	0.0003	0.0000
0.0003	0.0000	0.0000	0.0000	0.0000	0.0000	0.0000
0.0006	0.0002	0.0000	0.0000	0.0001	0.0000	0.0000
0.0008	0.0006	0.0001	0.0000	0.0000	0.0000	0.0000
0.0003	0.0000	0.0000	0.0000	0.0000	0.0000	0.0000
0.0000	0.0002	0.0003	0.0002	0.0001	0.0000	0.0000
0.0018	0.0003	0.0002	0.0001	0.0000	0.0000	0.0000
0.0006	0.0017	0.0012	0.0000	0.0001	0.0001	0.0001
0.0003	0.0000	0.0001	0.0000	0.0000	0.0001	0.0000
0.0006	0.0002	0.0000	0.0000	0.0001	0.0000	0.0000

F9	F10	F11	F12	F13	F14	F15
-0.4792	-0.1895	0.0513	0.0731	0.2220	-0.1354	0.2819
-0.3687	-0.2070	0.1227	0.1275	0.1724	-0.0601	0.2382
-0.4046	-0.0850	0.1337	0.2058	0.2702	-0.0780	0.2833
-0.0435	-0.0731	0.0647	0.0076	-0.0385	-0.1027	-0.2351
0.0250	-0.0986	-0.0262	0.0924	0.0032	-0.1018	-0.3132
0.1394	-0.0131	-0.0786	0.0278	-0.0884	-0.0471	-0.2177
0.0716	-0.0337	-0.0216	-0.0150	-0.1574	-0.0837	-0.2385
0.1180	0.0561	0.0064	-0.0024	-0.1655	-0.0974	-0.2168
0.1252	0.0900	-0.0305	-0.0476	-0.1820	-0.0174	-0.2539
0.0811	0.0938	-0.0257	-0.0504	-0.1728	0.0644	-0.1695
0.1073	-0.0221	-0.0395	-0.1397	-0.1950	0.0487	-0.1464
0.1410	0.1173	-0.0427	-0.0606	-0.2024	0.0918	-0.0940
-0.0362	-0.1036	0.0337	-0.0691	-0.0036	-0.0484	-0.1087
-0.1618	-0.0241	0.2305	0.0377	0.0745	-0.1550	-0.0928
-0.1213	-0.0567	0.2683	-0.0320	-0.0457	-0.0510	-0.0306
0.1023	0.0967	-0.0945	-0.0134	-0.2452	0.0937	-0.1454
0.0979	-0.0329	-0.0086	0.0319	-0.3070	0.1020	-0.1576

0.1295	0.0891	-0.0446	0.0038	-0.2236	0.1232	-0.1986
-0.0234	0.2349	-0.2551	-0.1424	0.0301	0.2274	0.0096
-0.0132	0.3281	-0.2222	-0.1535	-0.0351	0.1758	0.0334
0.0009	0.2234	-0.1906	-0.1310	-0.0611	0.2818	0.1077
0.2432	-0.1381	0.0131	0.1768	-0.0271	0.0627	0.3144
0.2985	-0.0371	0.0581	0.1845	0.0250	0.2293	0.2363
0.2868	-0.0774	0.0329	0.2179	0.0641	0.2359	0.3243
0.0826	-0.0009	-0.0633	-0.0605	-0.0729	-0.1073	-0.1543
-0.0816	0.0722	-0.0789	-0.0121	0.0682	-0.1125	-0.1808
0.2026	0.1756	0.0635	-0.1686	-0.1122	-0.0968	-0.1439
-0.1151	-0.0398	0.0077	-0.1197	0.2146	0.1033	0.3061
-0.0503	-0.1873	0.0161	-0.0602	0.1525	0.1692	0.2359
-0.0419	-0.0959	0.0192	-0.1115	0.2726	0.1553	0.2874
0.2668	-0.3584	-0.1770	-0.0885	0.3608	0.1516	0.5065
0.0691	-0.2329	-0.1444	-0.0858	0.4404	0.0963	0.4602
0.0128	-0.1564	-0.1919	-0.0353	0.4358	0.1234	0.5094
0.1633	-0.1847	-0.1553	0.1849	0.4130	0.0171	0.6756
0.1398	-0.0145	-0.0643	0.0731	0.4616	0.1012	0.5287
0.1055	0.0292	0.0462	0.1079	0.4659	0.1177	0.6522
0.1742	0.1584	-0.0140	-0.0497	-0.1749	-0.1167	-0.2722
0.1314	0.1058	0.0170	-0.0281	-0.1516	-0.0671	-0.2402
0.1941	0.0661	0.0875	-0.0338	-0.2111	-0.0507	-0.1792
-0.2619	0.0806	0.0754	0.0912	0.1550	-0.0585	0.2099
-0.4130	-0.0681	0.0690	0.1280	0.0844	-0.1164	0.0934
-0.3955	0.0725	0.0408	0.0384	-0.0052	-0.2385	0.1362
-0.1243	-0.1523	-0.0551	-0.0055	-0.1300	-0.0987	-0.3878
-0.1439	-0.0432	-0.0734	-0.0744	-0.2369	-0.2086	-0.4150
-0.0855	0.0282	0.0117	-0.0019	-0.2509	-0.2249	-0.4192
0.0471	0.1848	0.1052	-0.0194	-0.2790	-0.1115	-0.4809
-0.0770	0.1854	0.3608	-0.0053	-0.3180	-0.0564	-0.5098
-0.1150	0.2379	0.1626	0.0067	-0.2934	-0.1286	-0.4287

F9	F10	F11	F12	F13	F14	F15
55.4546	11.1951	1.1034	3.1110	30.5688	13.8043	99.2443
32.8172	13.3602	6.3001	9.4749	18.4467	2.7164	70.8153
39.5175	2.2511	7.4809	24.6849	45.2871	4.5849	100.2176
0.4572	1.6641	1.7531	0.0340	0.9216	7.9422	68.9971
0.1506	3.0324	0.2881	4.9730	0.0063	7.8003	122.4782
4.6912	0.0536	2.5882	0.4507	4.8503	1.6734	59.1688
1.2366	0.3531	0.1950	0.1320	15.3640	5.2739	71.0031
3.3604	0.9825	0.0172	0.0033	16.9862	7.1419	58.6810
3.7824	2.5271	0.3907	1.3206	20.5510	0.2292	80.4611
1.5875	2.7411	0.2773	1.4816	18.5253	3.1209	35.8639
2.7802	0.1524	0.6548	11.3715	23.5828	1.7821	26.7604
4.7993	4.2925	0.7644	2.1393	25.4071	6.3381	11.0304
0.3162	3.3469	0.4766	2.7826	0.0079	1.7610	14.7559
6.3198	0.1807	22.2466	0.8279	3.4420	18.0847	10.7501
3.5537	1.0030	30.1405	0.5952	1.2976	1.9612	1.1691
2.5285	2.9135	3.7417	0.1039	37.2874	6.6024	26.3798
2.3122	0.3369	0.0312	0.5935	58.4615	7.8316	31.0288
4.0467	2.4770	0.8319	0.0086	31.0222	11.4193	49.2599
0.1317	17.2023	27.2544	11.8187	0.5619	38.9437	0.1151
0.0419	33.5723	20.6775	13.7404	0.7652	23.2651	1.3957

0.0002	15.5670	15.2164	10.0000	2.3135	59.8009	14.4868
14.2811	5.9454	0.0724	18.2253	0.4568	2.9581	123.3903
21.5148	0.4291	1.4125	19.8462	0.3886	39.5697	69.7369
19.8583	1.8676	0.4525	27.6892	2.5458	41.8813	131.3338
1.6488	0.0002	1.6780	2.1351	3.3000	8.6717	29.7075
1.6070	1.6268	2.6060	0.0852	2.8882	9.5330	40.8111
9.9095	9.6133	1.6890	16.5667	7.8063	7.0582	25.8451
3.1993	0.4938	0.0249	8.3520	28.5680	8.0403	116.9523
0.6099	10.9392	0.1081	2.1103	14.4351	21.5419	69.4911
0.4232	2.8700	0.1550	7.2417	46.1152	18.1454	103.1039
17.1831	40.0581	13.1144	4.5672	80.7769	17.2981	320.2769
1.1515	16.9084	8.7299	4.2904	120.3451	6.9837	264.4752
0.0393	7.6240	15.4251	0.7285	117.8118	11.4621	324.0345
6.4398	10.6342	10.1001	19.9394	105.8148	0.2199	569.8614
4.7182	0.0657	1.7305	3.1134	132.2163	7.7067	348.9941
2.6869	0.2653	0.8945	6.7936	134.6855	10.4305	531.1013
7.3282	7.8199	0.0822	1.4373	18.9707	10.2450	92.5432
4.1692	3.4889	0.1216	0.4591	14.2500	3.3899	72.0117
9.1007	1.3643	3.2067	0.6668	27.6561	1.9374	40.0833
16.5611	2.0241	2.3789	4.8484	14.9089	2.5752	54.9931
41.1827	1.4474	1.9958	9.5548	4.4223	10.2022	10.8985
37.7759	1.6397	0.6981	0.8600	0.0167	42.8254	23.1648
3.7307	7.2359	1.2735	0.0175	10.4863	7.3293	187.7336
5.0013	0.5810	2.2533	3.2262	34.8249	32.7685	214.9977
1.7646	0.2478	0.0577	0.0022	39.0647	38.0894	219.4517
0.5365	10.6529	4.6329	0.2197	48.2825	9.3632	288.7597
1.4317	10.7216	54.5026	0.0163	62.7558	2.3980	324.5464
3.1957	17.6495	11.0752	0.0263	53.4191	12.4542	229.5183

F9	F10	F11	F12	F13	F14	F15
0.0069	0.0011	0.0001	0.0002	0.0015	0.0006	0.0024
0.0044	0.0014	0.0005	0.0005	0.0010	0.0001	0.0018
0.0059	0.0003	0.0006	0.0015	0.0026	0.0002	0.0029
0.0001	0.0003	0.0002	0.0000	0.0001	0.0006	0.0029
0.0000	0.0005	0.0000	0.0005	0.0000	0.0006	0.0055
0.0012	0.0000	0.0004	0.0000	0.0005	0.0001	0.0029
0.0004	0.0001	0.0000	0.0000	0.0019	0.0005	0.0043
0.0012	0.0003	0.0000	0.0000	0.0024	0.0008	0.0041
0.0014	0.0007	0.0001	0.0002	0.0030	0.0000	0.0059
0.0007	0.0010	0.0001	0.0003	0.0034	0.0005	0.0033
0.0013	0.0001	0.0002	0.0023	0.0045	0.0003	0.0025
0.0026	0.0018	0.0002	0.0005	0.0054	0.0011	0.0012
0.0001	0.0005	0.0001	0.0002	0.0000	0.0001	0.0006
0.0013	0.0000	0.0026	0.0001	0.0003	0.0012	0.0004

0.0008	0.0002	0.0039	0.0001	0.0001	0.0001	0.0001
0.0021	0.0019	0.0018	0.0000	0.0121	0.0018	0.0043
0.0022	0.0002	0.0000	0.0002	0.0217	0.0024	0.0057
0.0045	0.0022	0.0005	0.0000	0.0136	0.0041	0.0107
0.0006	0.0587	0.0693	0.0216	0.0010	0.0551	0.0001
0.0003	0.1622	0.0744	0.0355	0.0019	0.0466	0.0017
0.0000	0.0695	0.0506	0.0239	0.0052	0.1107	0.0162
0.0248	0.0080	0.0001	0.0131	0.0003	0.0016	0.0415
0.0393	0.0006	0.0015	0.0150	0.0003	0.0232	0.0246
0.0363	0.0026	0.0005	0.0210	0.0018	0.0246	0.0464
0.0005	0.0000	0.0003	0.0003	0.0004	0.0008	0.0017
0.0005	0.0004	0.0005	0.0000	0.0004	0.0010	0.0025
0.0034	0.0026	0.0003	0.0024	0.0011	0.0008	0.0017
0.0061	0.0007	0.0000	0.0066	0.0211	0.0049	0.0429
0.0011	0.0147	0.0001	0.0015	0.0097	0.0120	0.0233
0.0006	0.0030	0.0001	0.0041	0.0246	0.0080	0.0273
0.0071	0.0129	0.0031	0.0008	0.0130	0.0023	0.0257
0.0004	0.0048	0.0018	0.0007	0.0172	0.0008	0.0188
0.0000	0.0020	0.0031	0.0001	0.0158	0.0013	0.0216
0.0023	0.0029	0.0021	0.0029	0.0146	0.0000	0.0390
0.0015	0.0000	0.0003	0.0004	0.0164	0.0008	0.0215
0.0008	0.0001	0.0002	0.0009	0.0159	0.0010	0.0313
0.0032	0.0027	0.0000	0.0003	0.0032	0.0014	0.0079
0.0020	0.0013	0.0000	0.0001	0.0026	0.0005	0.0066
0.0045	0.0005	0.0009	0.0001	0.0054	0.0003	0.0039
0.0023	0.0002	0.0002	0.0003	0.0008	0.0001	0.0015
0.0053	0.0001	0.0001	0.0005	0.0002	0.0004	0.0003
0.0046	0.0002	0.0000	0.0000	0.0000	0.0017	0.0005
0.0003	0.0005	0.0001	0.0000	0.0004	0.0002	0.0033
0.0004	0.0000	0.0001	0.0001	0.0012	0.0009	0.0036
0.0001	0.0000	0.0000	0.0000	0.0012	0.0010	0.0034
0.0000	0.0006	0.0002	0.0000	0.0013	0.0002	0.0038
0.0001	0.0005	0.0020	0.0000	0.0016	0.0000	0.0040
0.0002	0.0008	0.0004	0.0000	0.0013	0.0002	0.0027

st

; FW
